# Pimozide Inhibits the Human Prostate Cancer Cells Through the Generation of Reactive Oxygen Species

**DOI:** 10.3389/fphar.2019.01517

**Published:** 2020-01-16

**Authors:** Ukjin Kim, C-Yoon Kim, Ji Min Lee, Bokyeong Ryu, Jin Kim, Changsoo Shin, Jae-Hak Park

**Affiliations:** ^1^ Department of Laboratory Animal Medicine, Research Institute for Veterinary Science, BK21 PLUS Program for Creative Veterinary Science Research, College of Veterinary Medicine, Seoul National University, Seoul, South Korea; ^2^ Department of Stem Cell Biology, School of Medicine, Konkuk University, Seoul, South Korea; ^3^ Department of Energy Resources Engineering, Seoul National University, Seoul, South Korea

**Keywords:** pimozide, drug repurposing, prostate neoplasm, reactive oxygen species, TRAMP

## Abstract

The United States Food and Drug Administration-approved antipsychotic drug, pimozide, has anticancer activities. However, the role of reactive oxygen species (ROS) in its effect on prostate cancer is not well-known. We examined cell proliferation, colony formation, migration, ROS production, and the expression of antioxidant-related genes after treatment of human prostate cancer PC3 and DU145 cells with pimozide. In addition, histopathology, ROS production, and superoxide dismutase (SOD) activity were analyzed after administering pimozide to TRAMP, a transgenic mouse with prostate cancer. Pimozide increased the generation of ROS in both cell lines and inhibited cell proliferation, migration, and colony formation. Oxidative stress induced by pimozide caused changes in the expression of antioxidant enzymes (SOD1, peroxiredoxin 6, and glutathione peroxidase 2) and CISD2. Co-treatment with glutathione, an antioxidant, reduced pimozide-induced ROS levels, and counteracted the inhibition of cell proliferation. Administration of pimozide to TRAMP mice reduced the progression of prostate cancer with increased ROS generation and decreased SOD activity. These results suggest that the antipsychotic drug, pimozide, has beneficial effects in prostate cancer *in vivo* and *in vitro*. The mechanism of pimozide may be related to augmenting ROS generation. We recommend pimozide as a promising anticancer agent.

## Introduction

New strategies in drug discovery are needed for more reliable drug development (Boguski et al., 2009). Among these strategies, drug repurposing is important to identify new applications of drugs that are already approved or abandoned (Carley, 2005a; Carley, 2005b). Drug repurposing in cancer treatment has successfully identified new uses of existing drugs and achieved significant results (Blatt and Corey, 2013). For example, metformin, a diabetic drug, has been reported to have anticancer activity in prostate cancer and several clinical trials are underway (Whitburn et al., 2017). Disulfiram, a drug for alcoholism, has also been reported to inhibit prostate cancer cell growth (Lin et al., 2011). Imatinib, developed to treat chronic myelogenous leukemia, is used to treat gastrointestinal stromal tumors (Saponara et al., 2014) and colorectal cancer (Kelley et al., 2013), which share a similar target.

Prostate cancer is the most commonly diagnosed non-skin cancer in men in developed countries and is a major cause of cancer-related deaths (Torre et al., 2015). In 2019, 174,650 men in the United States are expected to be diagnosed with prostate cancer and 31,620 will die from the diseases (Siegel et al., 2019). Prostate cancer is closely related to age, and several signaling pathways involving reactive oxygen species (ROS), which increase with age, play important roles in the development, and progression of cancer (Khandrika et al., 2009). In general, ROS promote cell proliferation, invasion, and metastasis, while inhibiting apoptosis, leading to cancer progression. Therefore, anticancer agents have been reported based on antioxidant effects (Adaramoye et al., 2015). However, higher levels of ROS have anti-cancer effects, by causing cell cycle arrest, apoptosis, and necrosis (Cairns et al., 2011).

Pimozide is a United States Food and Drug Administration-approved antipsychotic, and is used to treat Tourette syndrome and schizophrenia (Egolf and Coffey, 2014). Pimozide has shown anticancer effects in leukemia (Nelson et al., 2012), melanoma (Krummel et al., 1982), retinoblastoma (Bertolesi et al., 2002), breast cancer (Strobl et al., 1990), prostate cancer (Zhou et al., 2016), hepatocellular carcinoma (Fako et al., 2016), and osteosarcoma (Cai et al., 2017). The first reported anticancer effect of pimozide was that it acts as a dopamine antagonist in melanoma (Taub and Baker, 1979). It was also reported to inhibit STAT5 in leukemia (Nelson et al., 2012), and to function as a STAT3 inhibitor in prostate cancer (Zhou et al., 2016) and hepatocellular carcinoma (Chen et al., 2017). Recently, the ability of ROS generation to suppress osteosarcoma has been reported (Cai et al., 2017). However, the role of ROS in the anticancer effect of pimozide in prostate cancer is not well known. In this study, we demonstrated that pimozide affects prostate cancer cells *via* oxidative stress. Our work is the first study to provide empirical evidence that pimozide inhibits prostate cancer through generating ROS.

## Materials and Methods

### Reagents

Human prostate cancer cell lines PC-3 and DU145, and African green monkey kidney-derived Vero cell were acquired from the American Type Culture Collection (Manassas, VA, USA). Rat prostate cancer cell line AT-2 was obtained from Korean Cell Line Bank (KCLB, Seoul, South Korea). The non-tumorigenic human prostate epithelial cell line RWPE-1 was received from Dr. Won-Woo Lee (College of Medicine, Seoul National University, Seoul, South Korea). Normal prostate cell line WPMY-1 was received from Dr. So Yeong Lee (College of Veterinary Medicine, Seoul National University, Seoul, South Korea). PC-3, DU145, AT-2, and WPMY-1 cells were cultured in RPMI 1640 medium (Welgene, Gyeongsan, South Korea) supplemented with 10% fetal bovine serum (Gibco, Grand Island, NY, USA) and 1% penicillin/streptomycin (Gibco) at 37°C in 95% air/5% CO2. Vero cell was cultured in DMEM medium (Welgene) supplemented with 10% FBS and 1% PS at 37°C in 95% air/5% CO2. The RWPE-1 cells were cultured in keratinocyte serum-free medium (KSFM; Gibco) supplemented with 50 mg/L bovine pituitary extract and 5 *µ*g/L epidermal growth factor (EGF; Gibco). The Pimozide (Sigma, St. Louis, MO, USA) was dissolved in dimethyl sulfoxide (DMSO) (Duksan Pure Chemical Co., Ansan, South Korea) to obtain concentration of 25 mM. Diphenylene iodonium (DPI) (Sigma) was dissolved in DMSO to obtain concentration of 10 mM. The final DMSO concentration in the culture media was 0.1%. DMSO at the same final concentration of 0.1% was used as control. Glutathione (GSH) (Sigma) was dissolved in distilled water to obtain concentration of 100 mM. Same distilled water was used as control. The following antibodies were used: Mouse monoclonal anti-β-actin (A1976, Sigma, 1:1000), mouse monoclonal anti-SOD1 (Ab20926, Abcam, Cambridge, MA, USA, 1:1000).

### Cell Proliferation Assay

Cells (1.6 × 104) were seeded in 96-well plates in 200 *µ*[PLANE1] 4C1; media. The protocol for MTT assays was as described previously (Kim et al., 2019). The IC50 was calculated using computer program GraphPad Prism 5 (GraphPad Software, La Jolla, CA, USA) with nonlinear regression (log[inhibitor] vs. response - Variable slope).

### Clonogenic Assay

Diluted 1,000 cells were seeded with culture medium in six-well plates and cultured for 7 days. Colonies were stained with 1% crystal violet (Merck, Darmstadt, Germany) and at least 50 cells were counted.

### Scratch Assay

Cell motility was analyzed using an *in vitro* scratch assay. PC-3 and DU145 cells were seeded into 6-well cell culture plate (SPL) and grown to 90% or above confluence. Monolayers of prostate cells were then scratched using a pipette tip. The migration areas were measured using Image J software (https://imagej.nih.gov/ij/).

### ROS Measurement in Cell

The generation of intracellular ROS was determined using 2’,7’-dichlorofluorescin diacetate (DCFH-DA) (Sigma) which is converted to fluorescent 2’,7’-dichlorofluorescin in the presence of peroxides. After exposure to different concentrations of pimozide and GSH for 24 h, PC-3 and DU145 cells were treated with 10 μM DCFH-DA for 30 min at 37°C and washed with PBS. The cells were detached with trypsin-EDTA (Gibco), and intracellular ROS was detected using a fluorescence spectrometer Victor 3 (Perkin Elmer, Waltham, MA, USA) at 485 nm exposure and 535 nm emission.

### Real-Time Reverse Transcription-Polymerases Chain Reaction (PCR)

Total RNA was extracted using a Hybrid-R RNA extraction kit (GeneAll Biotechnology, Seoul, South Korea). cDNA was synthesized by M-MLV cDNA Synthesis kit (Enzynomics, Daejeon, South Korea) according to the supplier’s instructions. Quantitative real-time PCR was performed using TOPrealTM qPCR 2X PreMIX (Enzynomics) on a CFX Connect Real-Time PCR Detection system (Bio-Rad Laboratories, Hercules, CA, USA). Primers used were 5’-AGGGCATCATCAATTTCGAG-3’ (sense) and 5’-TGCCTCTCTTCATCCTTTGG-3’ (antisense) for human SOD1 (NCBI gene ID: 6647); 5’-GTGTGATGGTCCTTCCAACC-3’ (sense) and 5’-CTGACATCCTCTGGCTCACA-3’ (antisense) for human Prdx6 (NCBI gene ID: 9588); 5’-CAGTCTCAAGTATGTCCGT-3’ (sense) and 5’-AGGCTCAATGTTGATGGT-3’ (antisense) for human Gpx2 (NCBI gene ID: 2877); 5’-TTGGCTACCTTGCAGTTCGT-3’ (sense) and 5’-ATGTGAACCATCGCAGGCA-3’ (antisense) for human CISD2 (NCBI gene ID: 493856); 5’-CATGTACGTTGCTATCCAGGC-3’ (sense) and 5’-CTCCTTAATGTCACGCACGAT-3’ (antisense) for human β-actin (NCBI gene ID: 60). Ratio of target gene fold-change was normalized to human β-actin expression using comparative CT (2-ΔΔCt) method.

### Western Blot Analysis

The Cell lysates (20 *µ*g) were resolved by sodium dodecyl sulfate-polyacrylamide gel electrophoresis before transferring the proteins to nitrocellulose membranes and probing with mouse monoclonal anti SOD1 primary antibody and mouse monoclonal anti β-actin primary. Blots were then incubated with HRP-conjugated goat anti-mouse IgG secondary antibodies (Zymed, San Francisco, CA). The blots were developed with chemiluminescence using the Supersignal West Pico chemiluminescent substrate (Pierce, Rockford, IL, USA).

### Animals

The experimental protocol for animal handling was in accordance with the National Institute of Health (NIH) guidelines and approved by the Institutional Animal Care and Use Committee of Seoul National University (Protocol Number: SNU-181128-1). Male TRAMP mice expressing the SV40 large T-antigen under control of the prostate-specific rat probasin promoter were purchased from The Jackson Laboratory (Bar Harbor, ME, USA) and housed in the Animal Experiment Facility, College of Veterinary Medicine, Seoul National University. Mice were kept on a 12-hr light/dark cycle with ad libitum access to food and water. Pimozide was suspended in 10% DMSO, 10% Tween80 (Sigma), and 80% PBS (Biosesang, Seongnam, South Korea). The mice were randomly assigned to control and treatment groups (n = 5 for control and pimozide 10 mg/kg groups, and n = 3 for pimozide 5 mg/kg group). Pimozide at 5 and 10 mg/kg/5 times per week was administered by intraperitoneal injection to TRAMP males beginning at 12 weeks of age and was continued until the animals were 24 weeks old at which time the experiment was terminated. Body weight was measured weekly.

### Tissue Excision and Processing

At the time of sacrifice, the genitourinary tract (GUT: prostate, bladder, and seminal vesicle) were quickly excised and weighed. The anterior prostate lobes were dissected with inverted microscope (Olympus IX70), from one side and frozen in liquid nitrogen for ROS measurement and superoxide dismutase (SOD) assay. The remainder of each prostate was fixed in 10% buffered formalin and processed for standard paraffin sections.

### Histopathological Analysis

The mouse prostate were identified histopathologically in H&E-fixed sections using previously published criteria (Park et al., 2002; Berman-Booty et al., 2012). Briefly, we evaluated each prostate and assigned low-grade, moderate-grade, and high-grade prostate intraepithelial neoplasia (PIN), phyllodes-like tumor, well-differentiated adenocarcinoma, moderately differentiated adenocarcinoma, or poorly differentiated adenocarcinoma grade, based on the most severe lesion and most common lesions within the prostate. The distribution of lesions was also estimated as focal, multifocal, or diffuse. The distribution and lesion grade were then combined to calculate a distribution-adjusted histopathological score ranging from 0 to 42, which could be used for statistical analysis. Sections were examined in a blinded manner under light microscopy (Olympus AX70, Tokyo, Japan).

### ROS Measurement in Tissue

The quantitative measurement of ROS generation in prostate was performed with a slightly modified DCFH-DA method (Shao et al., 2012). The prostate samples were minced and homogenized in ice-cold PBS. The homogenates were centrifuged at 3,070 rpm for 10 min, the supernatants were re-centrifuged at 13,720 rpm for 20 min, and then the pellet, which contained mitochondria, was resuspended ice-cold PBS. All of manipulations above were carried out at 4°C. The DCFH-DA solutions at a final concentration of 2 μM were incubated for 30 min at 37°C. Fluorescence of samples was detected using a fluorescence spectrometer Victor 3 at 485 nm exposure and 535 nm emission.

### SOD Assay

Prostate tissue was dissected immediately and homogenized in ice-cold phosphate buffer. The homogenate was centrifuged, and the supernatant was used in the assay of SOD activity. SOD activity was determined according to the technical manual of the SOD assay kit-WST (Dojindo Molecular Technology Inc., Kumamoto, Japan).

### Statistical Analysis

All data are presented as mean ± standard error. Statistical significance (P < 0.05) was further analyzed with Student’s t-test using computer program GraphPad Prism 5.

## Results

### Pimozide Inhibits Cell Proliferation and Colony Formation in the PC3 and DU145 Cell Lines

**Figure 1:**
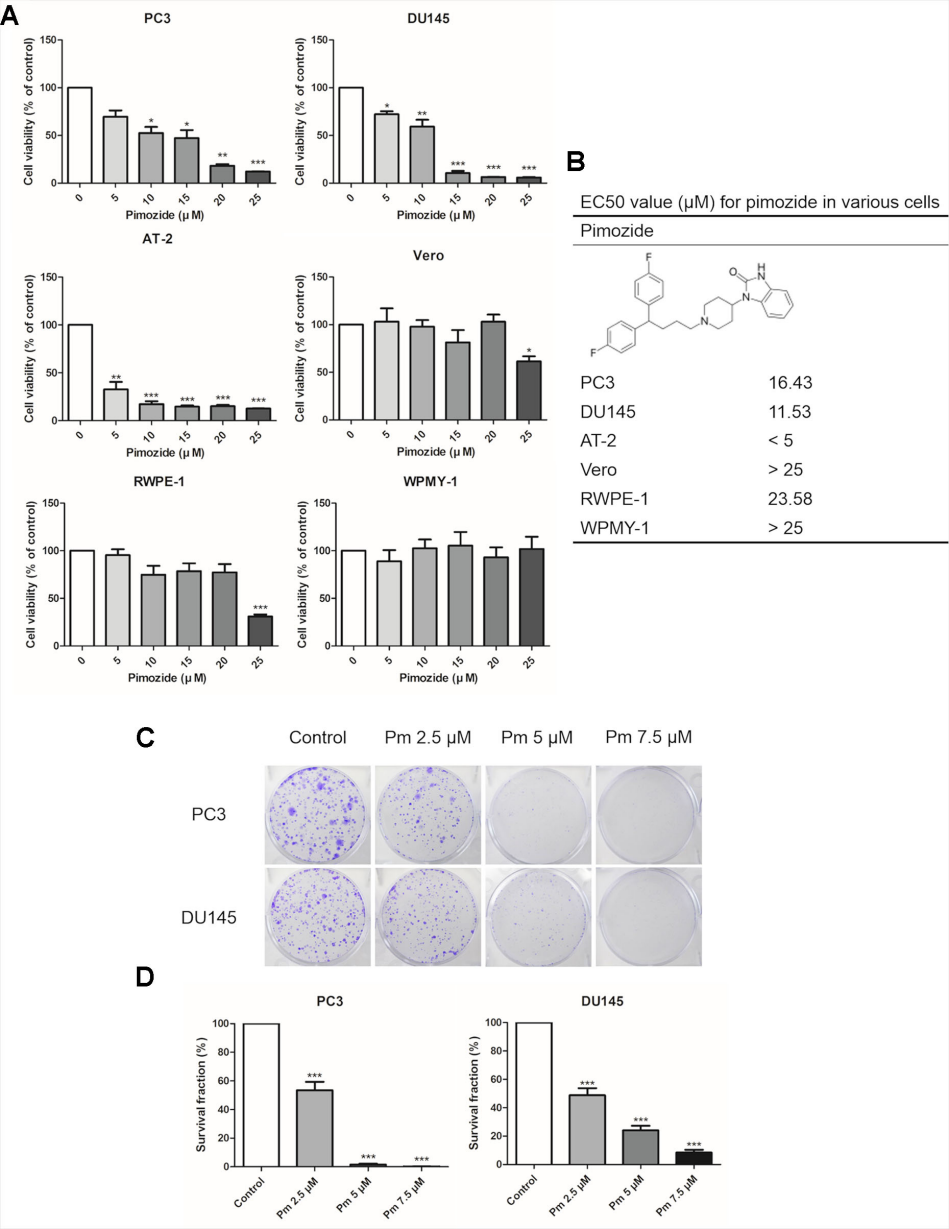
The effect of pimozide on cell proliferation. **(A)** The inhibition of proliferation of PC3, DU145, AT-2, Vero, RWPE-1, and WPMY-1 by pimozide was confirmed by MTT assay. **(B)** The structure of pimozide and estimated EC50 value for pimozide in various cells. **(C)** Colony formation of prostate cancer cell lines was inhibited in a concentration-dependent manner. **(D)** Surviving colony counts are plotted. *P < 0.05; **P < 0.01; ***P < 0.001. Results are presented as means ± SEM.

We analyzed the effect of pimozide on cell proliferation and colony formation. Treating human prostate cancer PC3 and DU145 cells, and rat prostate cancer AT-2 cells, with various concentrations of pimozide inhibited cell proliferation in a concentration-dependent manner (**Figure 1A**). As a negative control for prostate cancer, Vero cells (derived from the African green monkey) and normal prostate cell lines RWPE-1, and WPMY-1 were also treated with pimozide. There was no effect on the proliferation at concentrations below 20 μM. Based on these results, the half maximal effective concentrations (EC50) of pimozide were determined to be 16.43 μM, 11.53 μM, and 23.58 in PC3, DU145, and RWPE-1 cells, respectively (**Figure 1B**). Pimozide also inhibited colony formation in PC3 and DU145 cells (**Figure 1C**); surviving colony counts are plotted in **Figure 1D**.

### Pimozide Inhibits Migration of PC3 and DU145 Cells

**Figure 2:**
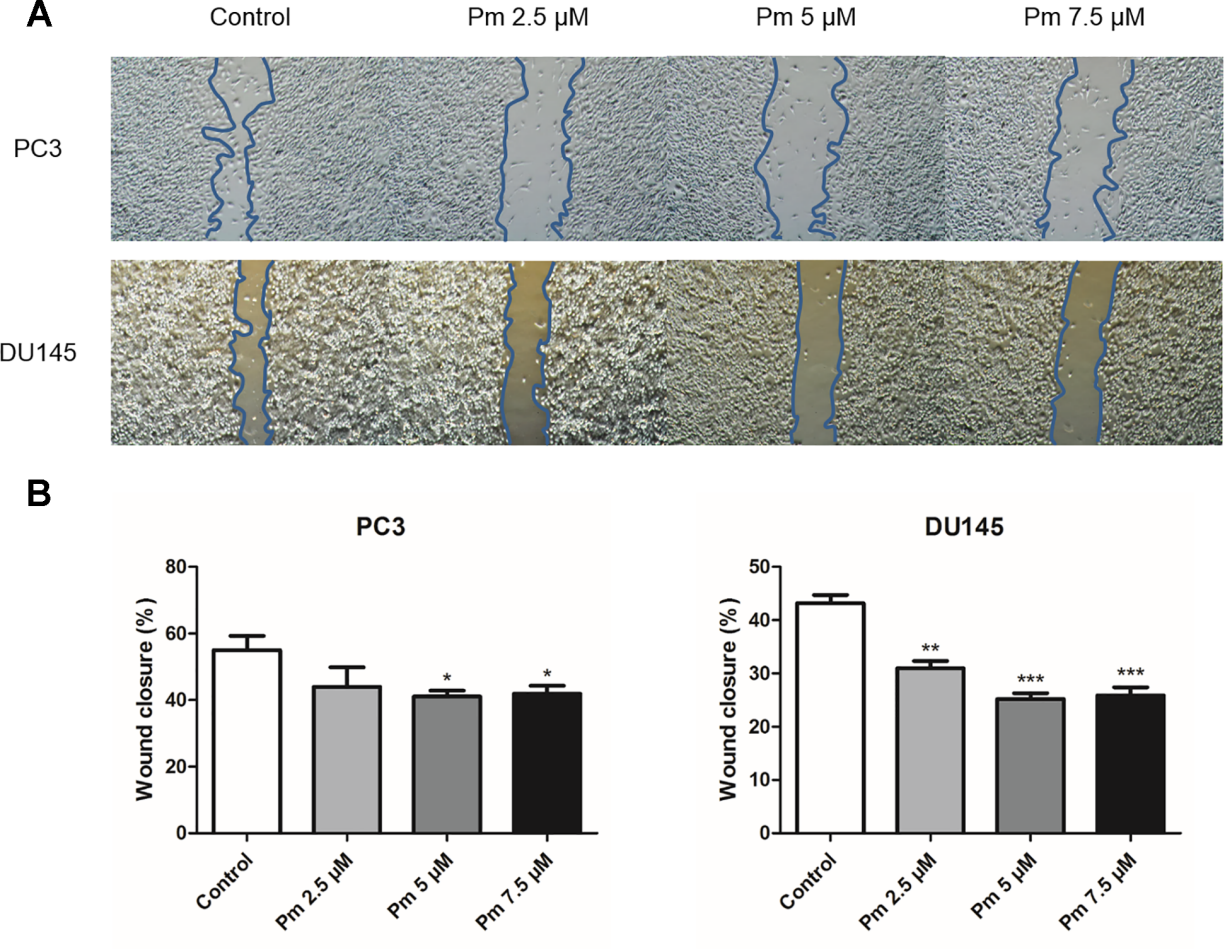
The effect of pimozide on migration of PC3 and DU145. **(A)** Pimozide suppressed the migration of PC3 and DU145 human prostate cancer cells. **(B)** Cells that migrated to the migration area were photographed (magnification, x40). *P < 0.05; **P < 0.01; ***P < 0.001. Results are presented as means ± SEM.

We evaluated the migration PC3 and DU145 cells treated with pimozide by the scratch assay. After a 24 h treatment with various concentrations of pimozide, cell migration into the scratched area was inhibited in a concentration-dependent manner (**Figure 2A**). The degree of migration was analyzed quantitatively and the results are shown in **Figure 2B**.

### Pimozide Inhibits the Proliferation of PC3 and DU145 Cells Through ROS Accumulation, Which Was Inhibited by the Antioxidant GSH

**Figure 3:**
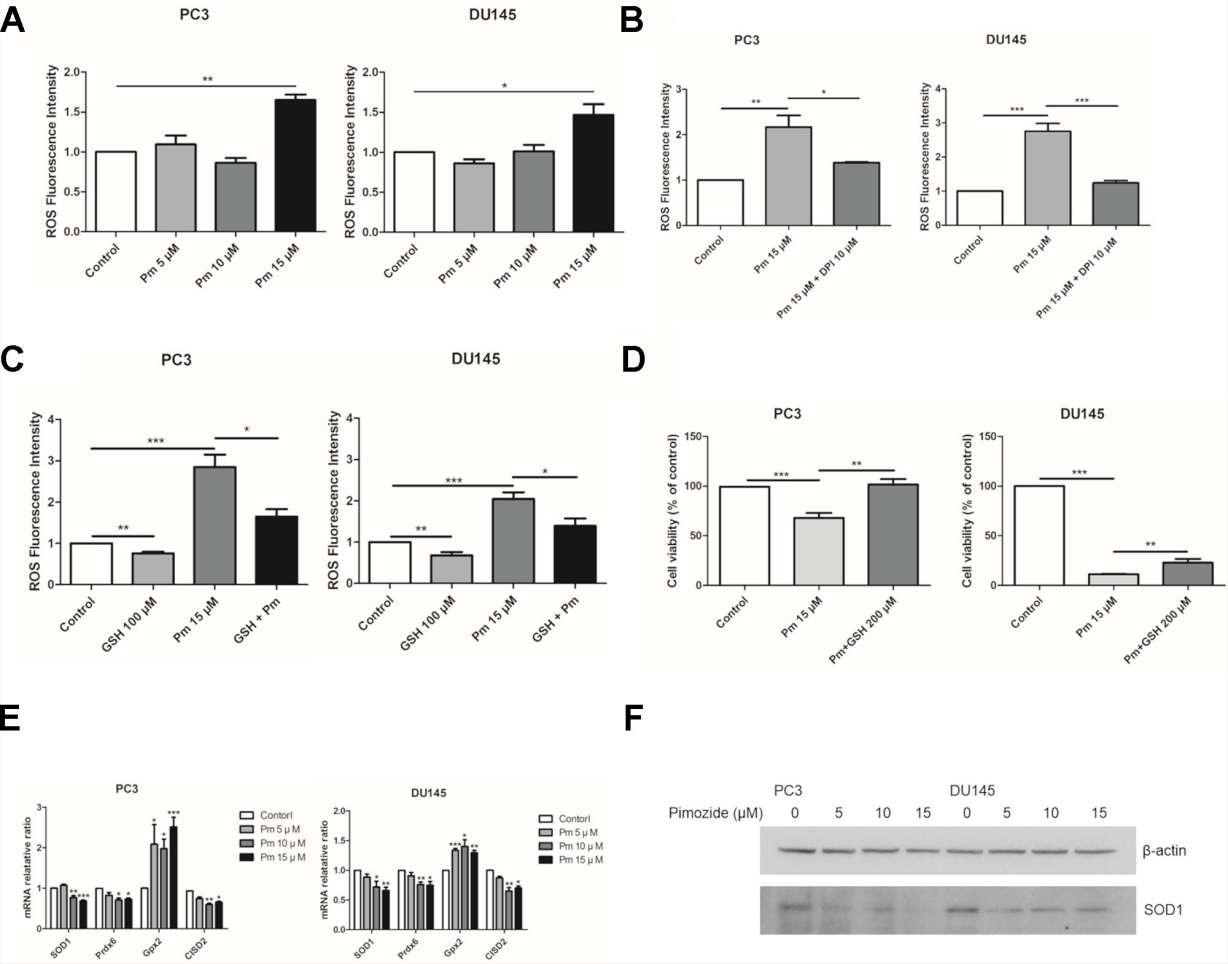
The effect of pimozide on the prostate cancer cells is related to ROS generation. **(A)** Pimozide induced generation of ROS in the PC3 and DU145 cells. Fluorescence spectrometer was used to determine ROS generation after staining with DCFH-DA. **(B)** The pimozide-induced ROS was inhibited by DPI. **(C)** ROS generation by pimozide was inhibited by the antioxidant GSH. **(D)** The inhibition of proliferation by pimozide was inhibited by the antioxidant GSH. Cell proliferation was confirmed by MTT assay. **(E)** Pimozide downregulated the mRNA expression levels of anti-oxidant enzymes SOD1, Prdx6, and upregulated the mRNA expression levels of anti-oxidant enzyme Gpx2 in PC3 and DU145 cells. The mRNA expression levels of CISD2 was downregulated in PC3 and DU145 cells. **(F)** Detection of the SOD1 protein expression by western blot in PC3 and DU145 cells. Pimozide decreased the protein expression levels of SOD1 in PC3 and DU145 cells. *P < 0.05; **P < 0.01; ***P < 0.001. Results are presented as means ± SEM.

ROS accumulation affects cell proliferation. We treated PC3 and DU145 cells with various concentrations of pimozide and measured ROS present in the cells after 24 h using the fluorescent dye, DCFH-DA. As expected, treatment of both cell lines with pimozide increased the production of ROS (**Figure 3A**). NADPH oxidase is known as a major source of ROS (D’Autréaux and Toledano, 2007). Treatment with DPI, an inhibitor of NADPH oxidase, decreased ROS production in pimozide-treated cancer cells (**Figure 3B**). We also investigated whether the production of ROS by pimozide was related to its ability to inhibit the proliferation of prostate cancer cells. When PC3 and DU145 cells were treated with 15 µM pimozide (which increased ROS production compared to the control group) and 100 µM GSH, ROS levels decreased compared with cells treated with pimozide alone (**Figure 3C**). Furthermore, the presence of 200 µM GSH reduced, to some extent, the pimozide-mediated inhibition of proliferation (**Figure 3D**).

The mRNA expression of SOD1, Prdx6, Gpx2, and CISD2 was analyzed after treatment of PC3 and DU145 cells with pimozide at concentration of 5, 10, and 15 μM for 24 h. Pimozide treatment reduced expression of the antioxidant enzymes SOD1, Prdx6, and CISD2 (which regulates the accumulation of ROS) (**Figures 3E**, **F**). However, mRNA expression of Gpx2 increased. Treatment of GSH with pimozide did not alter the expression of SOD1 and CISD2, but decreased Gpx2 expression (**Supplementary Figure 1**).

### Pimozide Inhibits Prostate Cancer Development in TRAMP Mice Through the Generation of ROS

**Figure 4:**
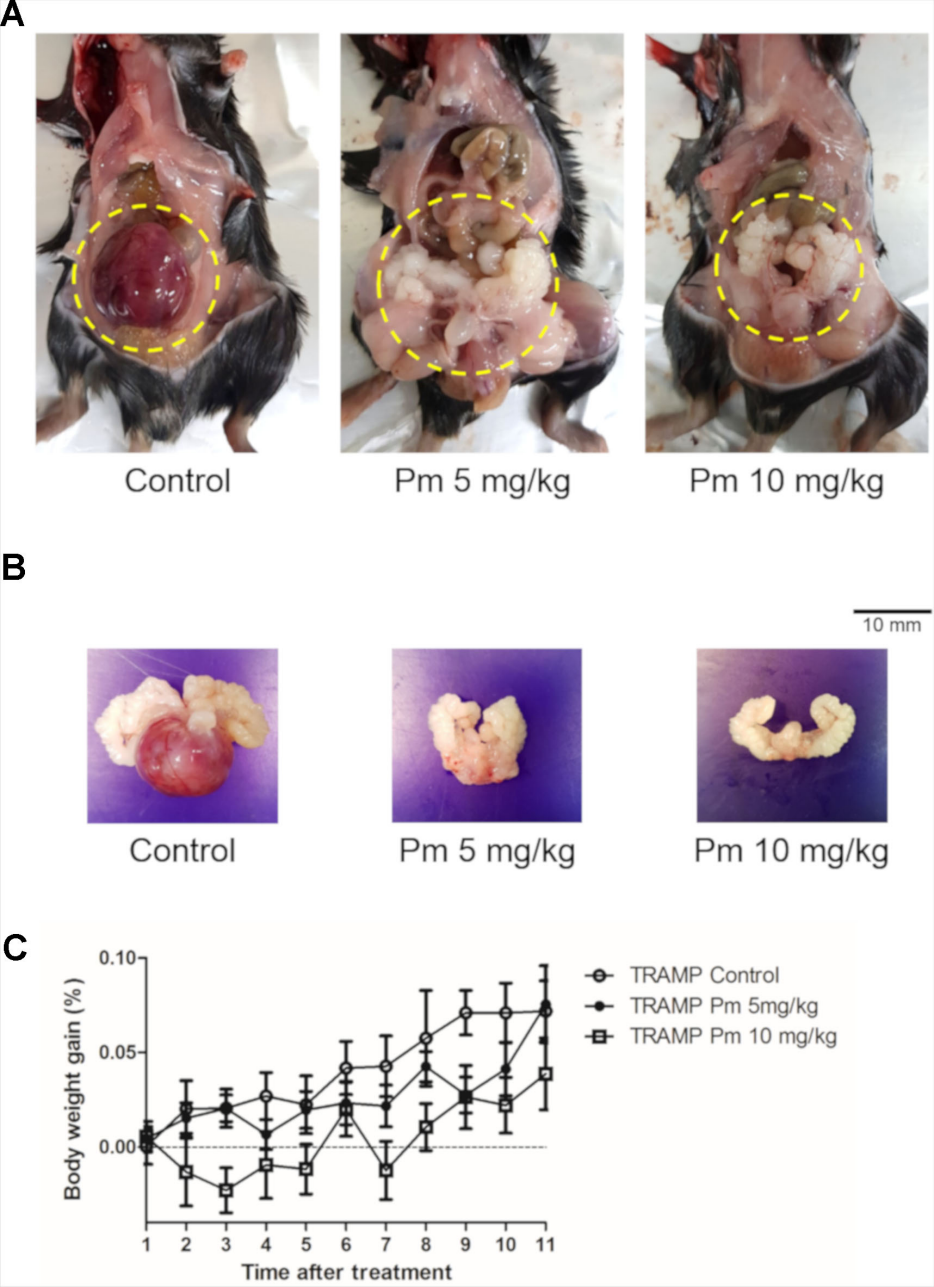
Effect of pimozide administration on prostate cancer progression in TRAMP mice. Pimozide was administered at 5 and 10 mg/kg/5 times per week by intraperitoneal injection beginning at 12 weeks of age for 12 weeks. **(A)** Representative pictures of dissected control and pimozide-treated TRAMP mice at 24 weeks of age. **(B)** Representative pictures of excised GUT from control and pimozide-treated TRAMP mice at 24 weeks of age. **(C)** Body weight gain profile of control and pimozide-treated TRAMP mice recorded weekly.

TRAMP mice show morphological and histological characteristics of human prostate cancer (Kaplan‐Lefko et al., 2003). We administered pimozide of 5 mg/kg and 10 mg/kg or vehicle to TRAMP mice and observed no noticeable toxicity or clinical symptoms during the treatment. All mice were necropsied after 12 weeks of treatment to grossly confirm the development of prostate cancer (**Figures 4A**, **B**). After 12 weeks of pimozide administration, there were no significant changes in body weight (**Figure 4C**).

**Figure 5:**
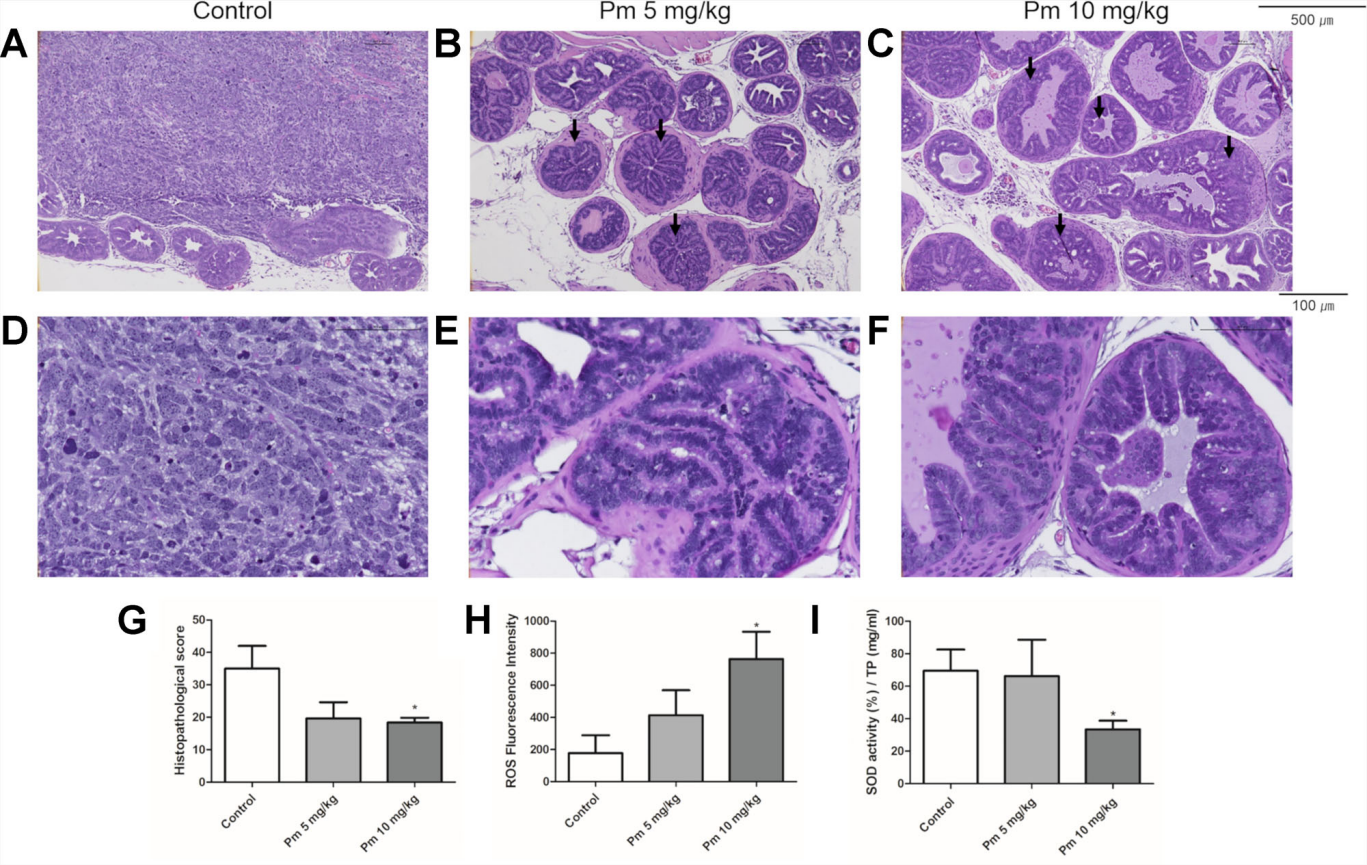
Effect of pimozide administration on prostate cancer progression in TRAMP mice. **(A** and **D)** Control TRAMP mice exhibited poorly differentiated adenocarcinoma with high mitotic index, frequent nuclear and cytoplasmic atypia, and the lack of glands (H&E, A x100; D x400). **(B**, **C**, **E**, and **F)** Administration of pimozide resulted in a marked reduction in adenocarcinoma and cribriform structure of epithelium with moderate and high-grade PIN (arrow) (H&E, B and C x100; E and F x400). **(G)** Histopathologic score was evaluated in TRAMP mice. Pimozide reduced histopathologic score. **(H)** Pimozide induced generation of ROS in the anterior lobes of prostate in TRAMP mice. **(I)** Pimozide inhibited the activity of anti-oxidant enzyme SOD in the anterior lobes of prostate in TRAMP mice. *P < 0.05. Results are presented as means ± SEM.

The prostate of TRAMP mice treated with pimozide was examined histopathologically. Representative histologic photographs are presented at magnifications of 100x and 400x in **Figures 5A**–**F**. In control untreated mice, poorly differentiated adenocarcinomas with irregular cytoplasm and nuclei were observed (**Figures 5A**, **D**). High-grade PIN (arrow), which almost completely filled the lumen of the prostate, was mainly observed in the 5 mg/kg pimozide group (**Figures 5B**, **E**). In the 10 mg/kg pimozide group, moderate-grade PIN (arrow) with epithelial cell proliferation was observed (**Figures 5C**, **F**). Histopathological scores were decreased in a dose-dependent manner in the pimozide-treated groups (**Figure 5G**). The ROS level, measured using DCFH-DA in the anterior lobe of the prostate, was higher in mice treated with pimozide than control mice (**Figure 5H**). SOD activity, measured in the anterior lobe of the prostate, was decreased in pimozide-treated mice compared to controls (**Figure 5I**).

## Discussion

Because of the limited success of current prostate cancer therapies, there is a growing demand for candidates for new prostate cancer treatments through drug repurposing. One possibility is pimozide, a United States Food and Drug Administration-approved antipsychotic drug that has therapeutic effects in many cancers (Nelson et al., 2012; Cai et al., 2017; Chen et al., 2017). In the current study, we report that pimozide inhibits the proliferation of PC3, DU145, and AT-2 prostate cancer cells. Importantly, this effect appears to be specific to cancer cells because the proliferation of Vero cells, a normal line derived from African green monkey (Prasannaraj et al., 2017; Kim et al., 2019), and normal prostate cell lines RWPE-1 and WPMY-1 was unaffected by pimozide at concentrations below 20 μM. This finding is consistent with a report that pimozide is specific for hepatocellular carcinoma (Fako et al., 2016).

We confirmed that the anticancer effect of pimozide was related to ROS generation as reported previously (Cai et al., 2017). Antioxidants have been reported to inhibit ROS-based anticancer effects (Xiao et al., 2010). Treatment with DPI, an inhibitor of NADPH oxidase, inhibited ROS production by pimozide. This means that NADPH oxidase is partially involved in ROS generation by pimozide. To determine whether ROS produced by pimozide were involved in its anticancer effect, GSH, an antioxidant, was administered. GSH decreased the ROS produced by pimozide and prevented the pimozide-mediated inhibition of proliferation of prostate cancer cells. The restoration of cell viability by GSH in presence of pimozide looks much weaker in DU145 compared to PC3. PC3 is a prostate cancer cell line with higher malignancy than DU145, and this difference appears to be the results (Wu et al., 2013). This suggests that ROS play an important role in the anticancer mechanism of pimozide.

CISD2 belongs to the CDGH iron sulfur domain-containing family (Lill, 2009). CISD2 inhibits ROS production in lung cancer, which is reportedly associated with a poor prognosis of lung adenocarcinoma (Li et al., 2017). In the current study, pimozide reduced the expression of CISD2 and the antioxidant enzymes SOD1 and Prdx6, which might have augmented ROS levels. In contrast, the expression of Gpx2 increased and GSH treatment decreased the expression. This may be because, under oxidative stress conditions, Nrf2 increases the expression of antioxidant enzymes and Gpx2 is a target of Nrf2 (Jayakumar et al., 2014). Overall, pimozide increased the production of ROS in prostate cancer cells and inhibited their proliferation by overcoming the antioxidant enzyme capacity.

TRAMP mice are a suitable animal model of human prostate cancer. In this mouse, prostate cancer occurs spontaneously and shows the histological and molecular characteristics of human prostate cancer (Kaplan‐Lefko et al., 2003). We have shown that the histological scores were decreased in TRAMP mice treated with pimozide. In addition, it was demonstrated that there were no significant changes in body weight indicating clinical symptoms following treatment. There was no significant difference in the weight of GUT (unpublished data).

Through the experiments reported here, we confirmed that pimozide plays an important role in the inhibition of prostate cancer *in vitro* by producing ROS and, importantly, that this effect can be reproduced *in vivo*. Increased ROS production was observed in the anterior prostate of pimozide-treated mice. We also confirmed that the activity of SOD, an antioxidant enzyme, was decreased. Based on these results, we hypothesized that pimozide formed ROS in TRAMP mice and decreased the activity of antioxidant enzymes. Thus, the ROS level exceeded the ability of prostate cancer cells to remove them and inhibited the cancer.

These results may be interpreted as contradictory to previous reports that ROS induce prostate cancer. However, it is necessary remember that ROS play different roles at different levels and in different stages of the tumor (Poillet-Perez et al., 2015). For example, ROS can induce DNA damage at early times to promote cancer initiation, but higher levels of ROS can induce apoptosis thereby inhibiting cancer (Kim et al., 2016). In the control group with prostate cancer, the ROS level was lower than in the pimozide-treated group without prostate cancer, suggesting that above a certain level ROS inhibited cancer. However, the level of ROS in the control group is expected to be higher than that of non-transgenic littermates that do not develop prostate cancer.

The results of this study suggest that pimozide has a therapeutic effect on prostate cancer *in vivo* and *in vitro*. The mechanism by which pimozide inhibits prostate cancer appears to be associated with increased ROS production. Co-treatment with pimozide and the antioxidant, GSH, decreased ROS levels and the anticancer effect of pimozide. *In vivo*, prostate cancer was reduced in TRAMP mice treated with pimozide, and this effect was associated with increased ROS. Thus, we suggest pimozide as a promising anticancer therapy agent.

## Data Availability Statement

The raw data supporting the conclusions of this article will be made available by the authors, without undue reservation, to any qualified researcher.

## Ethics Statement

The animal study was reviewed and approved by the Institutional Animal Care and Use Committee of Seoul National University.

## Author Contributions

J-HP and CS conceived and designed the experiments and J-HP is GUARANTOR for the article. UK, C-YK, JL, BR, and JK performed experiments. UK and C-YK analyzed the data and wrote the manuscript.

## Funding

This study was supported by the NRF (National Research Foundation of Korea) grant funded by the Korean government (NRF-2017-Global Ph.D. Fellowship Program) and partially supported by the Research Institute for Veterinary Science, BK21 PLUS Program for Creative Veterinary Science Research, Seoul National University.

## Conflict of Interest

The authors declare that the research was conducted in the absence of any commercial or financial relationships that could be construed as a potential conflict of interest.

## Supplementary Material

Supplementary Figure 1 The Effect of pimozide and GSH on mRNA expression levels of SOD1, CISD2, and Gpx2. Treatment of GSH with pimozide did not alter the expression of SOD1 and CISD2, but decreased Gpx2 expression. *P < 0.05; **P < 0.01; ***P < 0.001. Results are presented as means ± SEM.
