# Supplementary figures and images for: Pimozide Inhibits the Human Prostate Cancer Cells Through the Generation of Reactive Oxygen Species
Source: Front Pharmacol. 2020 Jan 16;10:1517. doi: 10.3389/fphar.2019.01517 (PMC6976539; doi:10.3389/fphar.2019.01517)

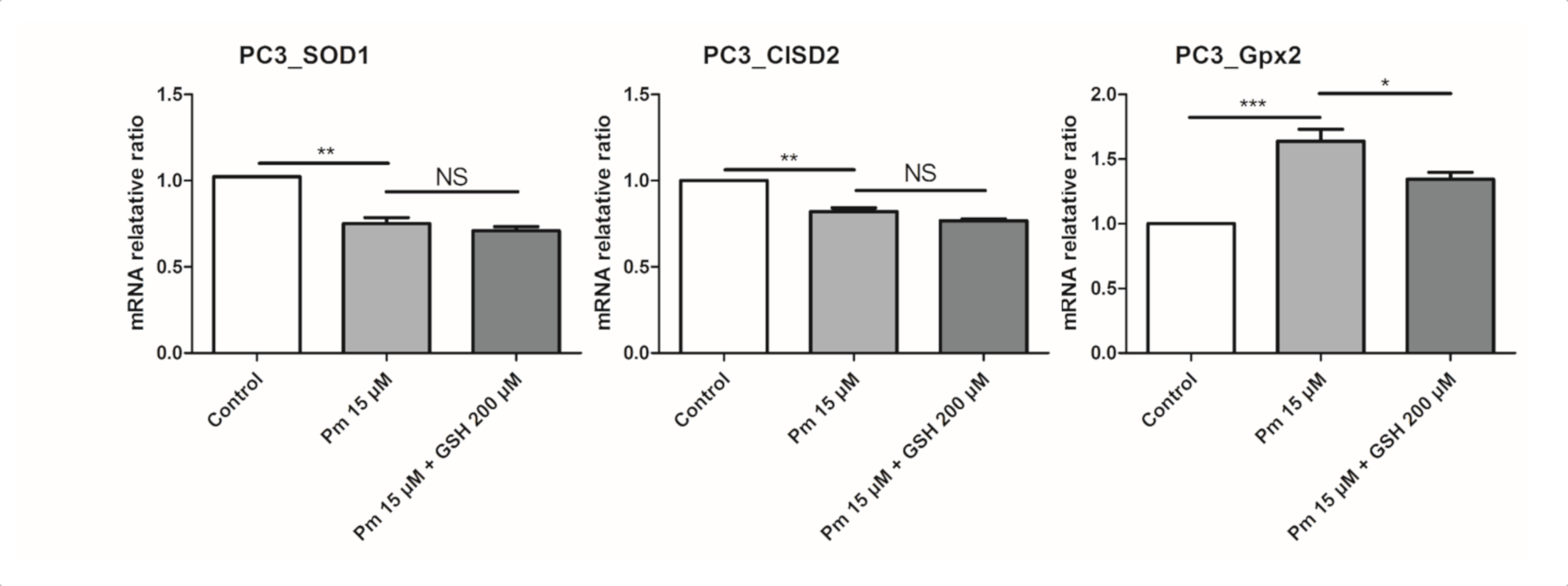

Supplement: Supplementary Figure 1 — The Effect of pimozide and GSH on mRNA expression levels of SOD1, CISD2, and Gpx2. Treatment of GSH with pimozide did not alter the expression of SOD1 and CISD2, but decreased Gpx2 expression. *P < 0.05; **P < 0.01; ***P < 0.001. Results are presented as means ± SEM. [file Image_1.tif]
